# The Coral Triangle and Strait of Malacca are two distinct hotspots of mangrove biodiversity

**DOI:** 10.1038/s41598-023-42057-6

**Published:** 2023-09-22

**Authors:** Tricia C. Goulding, Benoît Dayrat

**Affiliations:** 1 Department of Biology, Pennsylvania State University, University Park, PA 16802 USA; 2 Present Address: Department of Invertebrate Zoology, Smithsonian Institution, National Museum of Natural History, Washington, DC 20013 USA

**Keywords:** Biodiversity, Biogeography

## Abstract

Knowledge of the biogeography of marine taxa has lagged significantly behind terrestrial ecosystems. A hotspot of marine biodiversity associated with coral reefs is known in the Coral Triangle of the Indo-West Pacific, but until now there was little data with which to evaluate broad patterns of species richness in the coastal fauna of ecosystems other than coral reefs. This data is critically needed for fauna with low functional redundancy like that of mangroves, that are vulnerable to habitat loss and rising sea levels. Here we show that the diversity of mangrove fauna is characterized by two distinct hotspots in the Indo-West Pacific, associated with two habitat types: fringe mangroves in the Coral Triangle, and riverine mangroves in the Strait of Malacca, between the west coast of Peninsular Malaysia and Sumatra. This finding, based on a family of slugs of which the systematics has been completely revised, illustrates an unexpected biogeographic pattern that emerged only after this taxon was studied intensively. Most organisms that live in the mangrove forests of Southeast Asia remain poorly known both taxonomically and ecologically, and the hotspot of diversity of onchidiid slugs in the riverine mangroves of the Strait of Malacca indicates that further biodiversity studies are needed to support effective conservation of mangrove biodiversity.

## Introduction

The world's highest diversity of marine species occurs in the coral reefs of a region of the Indo-West Pacific known as the Coral Triangle, encompassing central and eastern Indonesia, the Philippines, Papua New Guinea, and Melanesia^1–4^. Recent studies utilizing molecular data have revealed previously unrecognized species from the Coral Triangle^5–7^ and provided an even greater resolution to the geographic gradient in species diversity in several groups such as corals, reef-associated algae, fishes, snails, and foraminifera^3,8–13^. Studies of gastropod biogeography in the Indo-West Pacific have focused on a few families that are well-known and abundant on coral reefs, the cone snails (Conidae), cowries (Cypraeidae), and volutes (Volutidae), and have pointed towards a pattern of high biodiversity in the Philippines and Melanesia, the northern and eastern edges of the Coral Triangle^8,9^. However, there remain many numerous other taxa in which species diversity and distribution are poorly known in the Indo-West Pacific, particularly in marine ecosystems other than shallow-water reefs, such as seagrass beds, mangroves, and the deep sea. The species unique to these marine ecosystems are vulnerable to multiple threats, including habitat loss and degradation^14,15^, rising sea levels^16–18^, pollution from agricultural and industrial sources^19,20^, and deep-sea mining^21^, but the lack of data on species diversity and distribution across the region prevents effective conservation and limits our understanding of the causes for biogeographic patterns. One way to address this issue is to investigate diversity gradients by mapping species distributions and estimating species richness of well-studied taxa across the Indo-West Pacific. However, this type of analysis relies on robust datasets in which (1) cryptic diversity has been investigated through integrative taxonomic studies, (2) species have been sampled evenly across the region, and (3) species distributions are known for a high proportion of species.

The diversification of marine taxa in the world’s oceans is influenced by a variety of factors, including abiotic conditions, habitat heterogeneity, land area, and the geologic history of the region^12,22–24^. Therefore, there is no reason to expect the biogeographic patterns observed in coral reefs, i.e., a hotspot of diversity centered in the Coral Triangle, to be consistent in other ecosystems for which all those factors may differ. Investigations of diversity gradients in corals, reef fishes, and various groups of invertebrates have suggested that biogeographic patterns may vary between taxa, but in most of these studies, the peak in species diversity is observed within the boundaries of the Coral Triangle^8,9,25^. Mangrove forests are a unique kind of coastal habitat found between land and sea in tropical and subtropical regions of the world. Mangrove plant diversity is by far the highest in the Indo-Malay Archipelago (i.e., the region including Indonesia and Malaysia) and Papua New Guinea, where dozens of plant species produce large forests influenced by both freshwater inputs and seawater tides^26–29^. The foundation of mangrove communities are the plants, which belong to multiple, unrelated lineages, and are defined by their physiological specialization to the salt-water environment and the taxonomic isolation of the family or genus from terrestrial relatives. The Avicenniaceae, Combretaceae, Palmae, Rhizophoraceae, and Sonneratiaceae are all major components of the flora of mangroves forests, which together with nearly a dozen minor groups, form a complex forest habitat for a variety of animals. These forests may also include mangrove associates such as ephiphytes and climbers, but these are usually present in the back of mangrove forests and play an inconspicuous role in their structure^30^. A relatively low diversity of plants occur in mangrove forests, but they are highly productive ecosystems that provide habitat to thousands of marine and terrestrial animals, including insects, mollusks, crustaceans, and vertebrates^31–34^. Over the last several decades, these ecosystems have been highly impacted, particularly by high rates of deforestation. More than 35% of mangrove forest area was lost globally in the 1980s and 1990s, with even higher losses in some regions^35,36^. While deforestation rates have slowed over the last 20 years, these forests still had the highest ratio of loss to gain of tidal wetlands globally between 1999 and 2019^37^. Recent data show that six of the ten countries with the highest rates of mangrove deforestation are in Southeast Asia, with Myanmar, Malaysia, India, and Indonesia topping the list^38^. Thus, understanding patterns of faunal diversity in the mangroves of Southeast Asia and Australia is critical in order to inform conservation efforts. However, patterns of species richness in mangrove invertebrates remain unclear in the Indo-West Pacific. The present contribution addresses whether the highest mangrove gastropod diversity is in the region of the Coral Triangle or elsewhere, and highlights possible reasons for observed differences.

Mangroves provide complex, heterogeneous habitats for many types of animals, but few groups of organisms have diversified in this ecosystem, which requires surviving long periods of submergence followed by exposure to air, freshwater inputs, high temperatures, highly variable salinity, and anaerobic soils, all of which are harsh environmental conditions for most plants and animals. Compared to the many thousands of gastropods and other invertebrates associated with coral reefs, only a few hundred species of gastropods have been recorded from mangrove forests^31,39^. Yet, despite moderate levels of diversity in mangrove forests, the species within them remain poorly known, particularly the invertebrates^32^. The gastropods that have adapted to mangroves belong primarily to two gastropod lineages: the pulmonate gastropods, a group of mostly terrestrial air-breathing snails, and caenogastropods, a group of snails which ordinarilly use a gill for respiration, in which some taxa have also adapted to breathe air^40–42^. Onchidiids are air-breathing pulmonate slugs and are not part of the opisthobranch sea slugs, which include well-known organisms such as nudibranchs and sea hares^43^. Because of their lung, onchidiids have diversified in upper-intertidal habitats where sea slugs like nudibranchs cannot survive. Onchidiid slugs feed on minute algae, diatoms, and biofilms, but also ingest sediment particles and detritus; a plesiomorphic mode of feeding common among gastropods^44,45^. Anatomically, onchidiid species display little variation between species, but species from different habitats (rocky intertidal vs. mangroves) and from different mangrove microhabitats (mud surface, tree roots and bark, etc.) are characterized by an intestine of different length, which is likely associated with the amount of time needed to extract nutrients from food mixed with different substrates^46^. Onchidiid species may also vary in developmental mode, with some species hatching from egg capsules and others developing through planktonic larvae, but this is known only in a few species, and it has been unclear how the biogeographic patterns in these intertidal slugs compare to other marine invertebrates^47–49^. The slugs of the family Onchidiidae are an ideal case study to investigate patterns of species diversity and distribution outside coral reefs in the Indo-West Pacific because this family has diversified in mangroves and rocky shores, and its systematics has recently been completely revised taxonomically and phylogenetically^46,50–62^. Onchidiid species diversity has been investigated based on even sampling effort by the authors worldwide and most especially across the entire Indo-West Pacific, from South Africa to Hawaii and from Japan to southeastern Australia. Further, each species was studied through a combination of DNA sequences and comparative anatomy to reveal any cryptic diversity. This revisionary work culminated in the description of six new genera and 25 new species representing 50% of the known species diversity in the Indo-West Pacific^46,50–62^.

## Results

**Figure 1:**
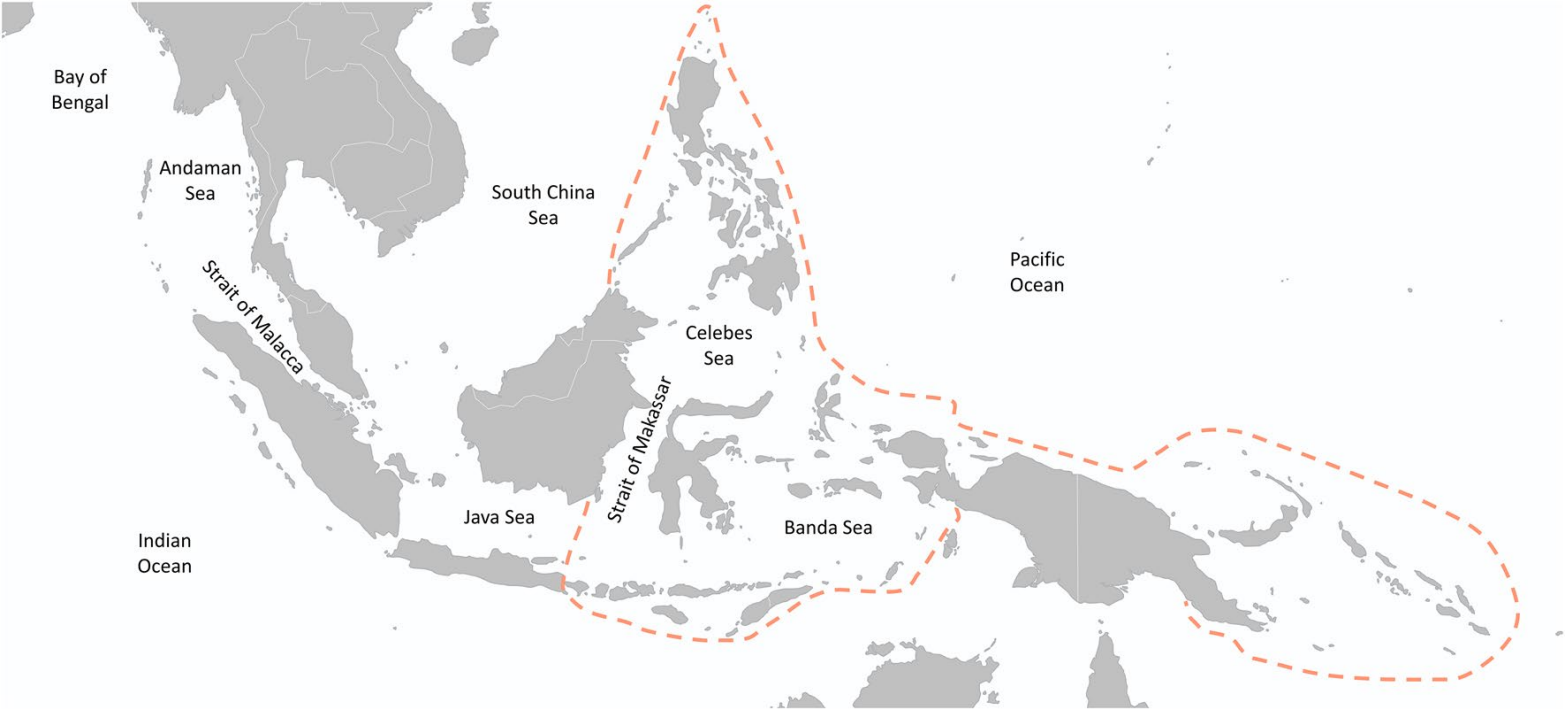
Map of the central Indo-West Pacific. The delimitation of the Coral Triangle is outlined based on Veron et al.^1^. Base map from Natural Earth (http://naturalearthdata.com).

**Figure 2:**
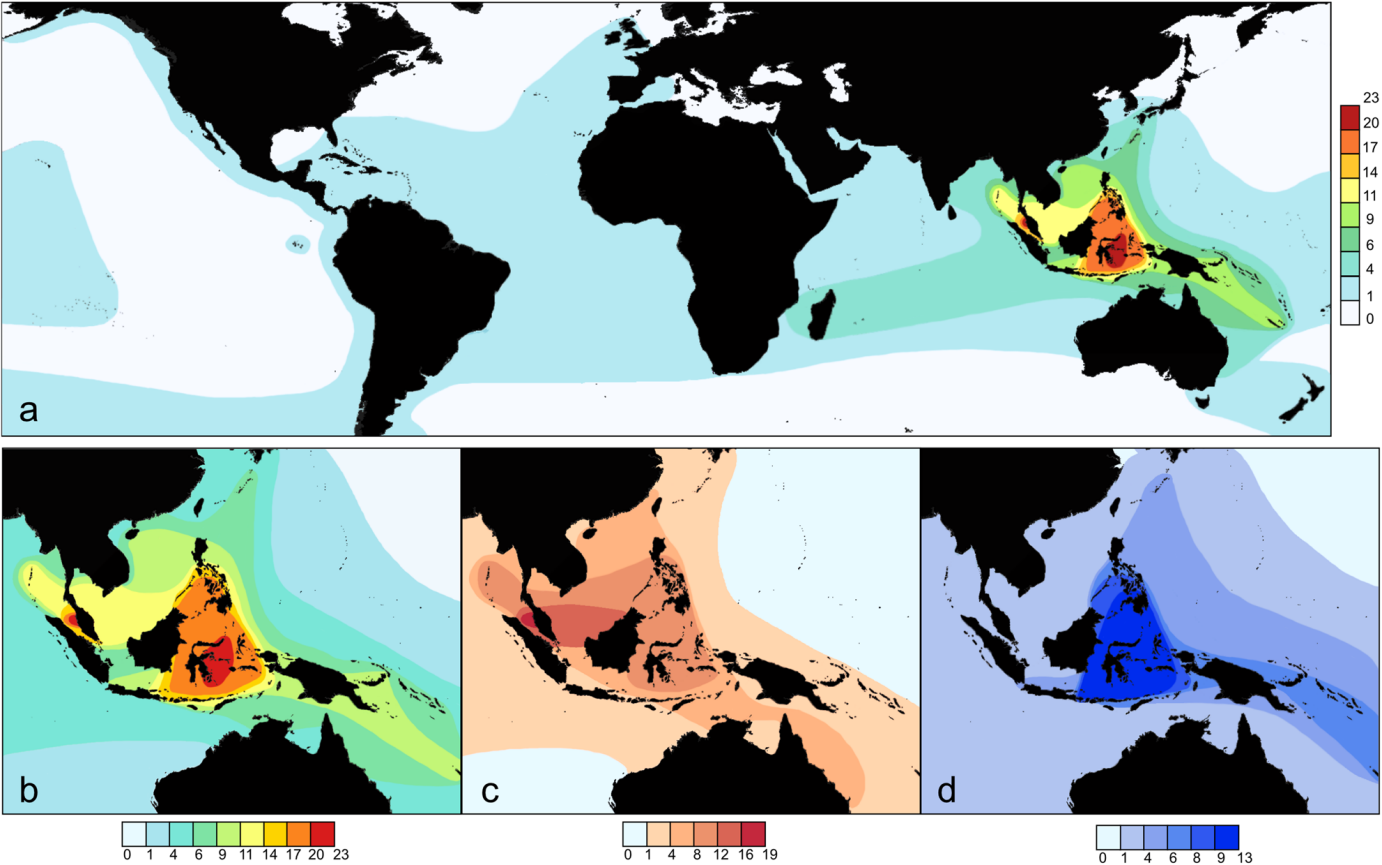
(**A**) Map of onchidiid species richness globally. Maps are based on GIS analysis of geographic distributions published in a series of taxonomic revisions^46,50–62^. (**B**) Focus on species richness of onchidiids in Southeast Asia. **(C**) Species richness of onchidiids associated with riverine mangroves. (**D**) Species richness of onchidiids associated with fringe mangroves, coral rubble and rocky intertidal habitats. See Supplementary Table 1 for list of species associated with each habitat type. Base map from Natural Earth (http://naturalearthdata.com).

Globally, the highest species diversity of onchidiid slugs occurs in the Indo-West Pacific, where they have diversified mostly in mangroves, compared to very low diversity in the Atlantic, Caribbean, and Eastern Pacific (Fig. 2A), where species are most commonly found in the rocky intertidal^46^. There is a steep cline in onchidiid species richness with distance from the Indo-Malay Archipelago, with low diversity on islands in the central Pacific and the western Indian Ocean, which is similar to the cline in mangrove plant diversity^63^. The steep cline in species richness in onchidiids is consistent with the dispersal limitation hypothesis and is similar to the cline in diversity observed in Erroneinae cowries with shorter planktonic durations^64^. However, and most interestingly, in the Indo-West Pacific, the highest onchidiid diversity occurs in two distinct regions: one peak of highest diversity is in Central Indonesia and the Philippines (i.e., in the Coral Triangle), and the other in the Strait of Malacca, between western Peninsular Malaysia and northern Sumatra (Figs. 1, 2B). The diversity of onchidiids is lower in Papua New Guinea and Melanesia, and decreases with distance, with only a single species present in the Hawaiian Archipelago.

The existence of two peaks of onchidiid diversity in the Indo-West Pacific reflects the fact that not all mangrove forests are the same. Indeed, the mangrove forests of the Indo-West Pacific differ in numerous characteristics, including sediment size, freshwater input, and plant species^28^. The diverse complexity of mangroves in the Indo-West Pacific makes them more difficult to classify than in the Caribbean and tropical America, but we can broadly distinguish between (1) riverine forests, which occur around river channels with nutrient-rich freshwater, and (2) fringe forests around coastal shorelines^65,66^. Natural riverine forests are deep, dense, and impenetrable, often with very tall trees, while fringe mangroves are narrower and open with much smaller trees. These forests overlap in their respective plant fauna but are characterized by differences in elevational slope relative to the tides, as well as sediment characteristics and other abiotic conditions^30^.

Another hypothesis that could be considered to explain the two peaks of diversity in onchidiid slugs is the influence of the tectonic history of the continents, which is reflected in the transition of terrestrial fauna and flora of Southeast Asian origin to those of Australian origin in the Indo-Malay Archipelago. Several variations of this boundary have been proposed for terrestrial taxa, most notably the Wallace Line along the Strait of Makassar between Bali and Lombok, and Huxley’s modification of it to move the line west of the Philippines, but also the Weber Line and Lydekker Line closer to the Sahul Shelf around Australia and Papua New Guinea^67,68^. However, there are few examples of a similar disjunction in coastal or other marine fauna, with most studies instead referring to intraspecific gene structure near this boundary, rather than a transition zone between different faunas^69–71^. This is likely because many marine species can be dispersed across the region with currents. If the tectonic history of the region exerted a strong influence on the geographic distributions of onchidiids, we would expect to find distinct phylogenetic groups associated with Australia and Papua New Guinea compared to those of Southeast Asia. A subset of this fauna would be expected to be present in the Coral Triangle, with fewer species crossing a biogeographic boundary. However, there is no onchidiid genus with more than one species that is particularly allied to Australia or Papua New Guinea, with only one monotypic genus possibly of Australian origin^56^. Rather, the onchidiid fauna of Australia is represented by a couple of species from multiple genera, with no genus being particularly well-represented. The biogeographic patterns observed in extant onchidiids (no onchidiid fossils are known)^72^ therefore does not seem to reflect a transition similar to the Wallace Line between fauna of Asian and Australian origin.

During the evolutionary history of the Onchidiidae, onchidiid slugs transitioned from rocky shores into coastal forests and diversified in these two types of mangroves^46^. Some adapted to distinct microhabitats within riverine mangrove forests, including silty muds (*Alionchis*, *Laspionchis*, *Paromoionchis*, and *Peronina*)^57,59,61,62^, mounds of mud and dead logs (*Onchidium*)^50,51^, roots and trunks of mangrove trees (*Melayonchis* and *Platevindex*)^52,54,55^, and supratidal fringes of mangroves (*Onchidina*)^56^. Other onchidiids diversified in coastal, fringe forests with sand and coral rubble (*Wallaconchis*)^58^ or transitioned back to the rocky intertidal (*Peronia, Marmaronchis*, and *Wallaconchis*)^53,58,60^. The association of onchidiids with rocky intertidal habitats is not phylogenetically constrained, with none of these three genera being sister-taxa, and all being highly diverged from the genus *Onchidella,* which also inhabits rocky shores at the margins of the Indo-West Pacific^46^. A similar distinction between species specialized to muddy, riverine mangroves and those specialized to coastal, fringe mangroves was observed in *Littoraria* snails (caenogastropod snails in the family Littorinidae) which were classified as either continental or oceanic, with oceanic species hypothesized to be less tolerant of habitats with muddy sediment^73^. This distinction extends outside mangrove forests, with differences also noted between taxa with continental and oceanic affinities in other coastal gastropods^74,75^.

**Figure 3:**
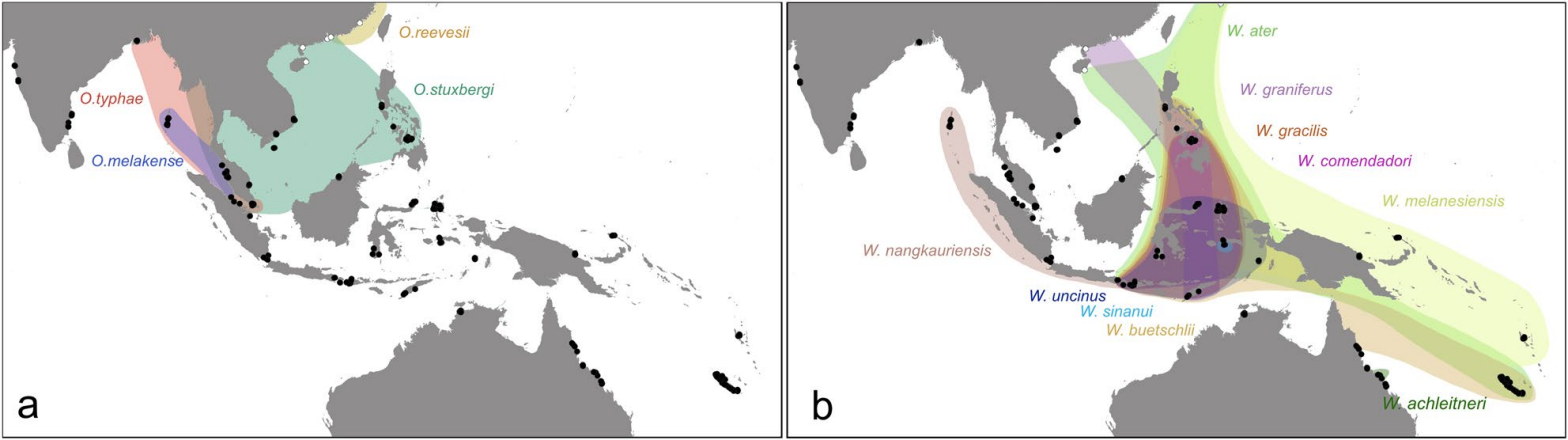
Map of species distributions in the genera. (**A**) *Onchidium.* (**B**) *Wallaconchis*. Sites in which onchidiid collections were sequenced in previous studies by the authors are marked with black dots and sequences from GenBank are marked with white dots^50,58^. Base map from Natural Earth (http://naturalearthdata.com).

A geographical comparison of the species richness for onchidiids living in riverine mangrove forests (Fig. 2C) with that of onchidiids living in fringe mangroves and rocky intertidal habitats (Fig. 2D) shows that it is the adaptation to those distinct habitats which causes the existence of two distinct peaks of diversity in the Indo-West Pacific (Fig. 2B). Onchidiids specialized to riverine mangroves are most diverse in the Strait of Malacca, as exemplified by the genus *Onchidium* (Fig. 3A), though other genera specialized to muddy mangroves have a broader geographic distribution extending to Australia and New Caledonia^52,61,62^. In contrast, onchidiids specialized to fringe mangroves with sand and coral rubble as well as the rocky intertidal are most diverse in central and eastern Indonesia and the Philippines, i.e., the Coral Triangle, with high diversity extending into Melanesia, which is largely due to a radiation of sympatric species in the genus *Wallaconchis* (Fig. 3B). These *Wallaconchis* species overlap in the same region with sympatric species of both *Marmaronchis* and *Peronia* to form a second peak of diversity that coincides with the high diversity of coral reef taxa in the Coral Triangle. The difference in the geographic distributions of *Onchidium* and *Wallaconchis* is striking, especially considering that there is geographic overlap in the two mangrove types in these regions. Fringe mangroves are not exclusive to the Coral Triangle region, but also occur in the Strait of Malacca and South China Sea, but only one *Wallaconchis* species is present in the fringe mangroves of that region. Riverine mangroves were also surveyed throughout the center of the Coral Triangle region including Sulawesi, an island with large riverine mangroves in the Coral Triangle, but a lower diversity of fauna was found in the riverine mangroves compared to the Strait of Malacca. To our knowledge, this is the first time that ecological adaptations within a clade of marine taxa are recognized to directly contribute to two distinct regions of high diversity. Two peaks of biodiversity were also proposed in a meta-analysis of mangrove crab diversity, one in the South China Sea between Singapore and Southwestern Indonesia, and a second in southern India^76^. However, in that case, temperature was proposed as the main driver of the diversity pattern.

**Figure 4:**
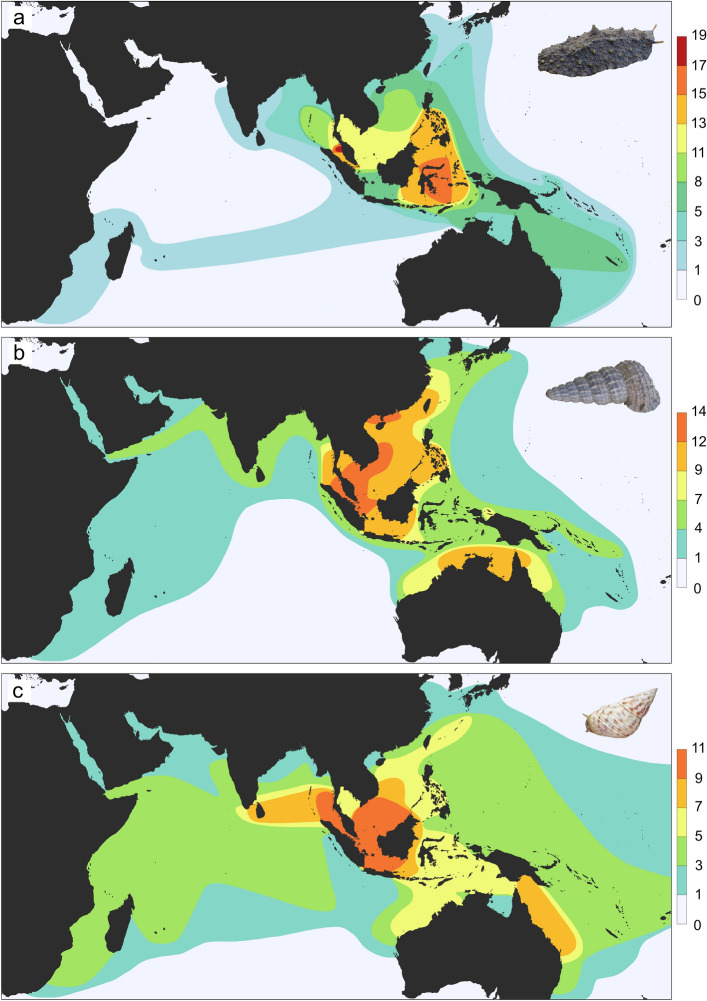
Patterns of species richness of mangrove gastropods in the Indo-West Pacific. Maps are based on GIS analysis of geographic records detailed in taxonomic and molecular phylogenetic studies. (**A**) Mangrove onchidiids (9 genera, 38 species). (**B**) Mangrove potamidids (5 genera, 38 species). (**C**) Mangrove littorinids (1 genus, 22 species). Base map from Natural Earth (http://naturalearthdata.com).

When the diversity of all mangrove onchidiids is considered (i.e., excluding onchidiids from rocky intertidal habitats), two peaks of species richness are observed (Fig. 4A), similar to the pattern observed from all onchidiids (Fig. 2B). The highest diversity of mangrove slugs is in the riverine mangroves in the Strait of Malacca, but a second peak with slightly fewer species occurs around Sulawesi, at the western side of the Coral Triangle. These patterns can be compared to two other groups of mangrove gastropods, which are found almost exclusively in mangroves. The Potamididae includes five genera of gastropods adapted to mangroves and the Littorinidae includes one genus that diversified in mangroves, the *Littoraria*. Both of these families belong to the Caenogastropoda, a group of snails deeply divergent from the pulmonates. Studies of *Littoraria* and potamidid mangrove snails have also suggested that these snails reach high diversity in the Strait of Malacca, but the existing map of species richness in *Littoraria* is outdated^77^ and no map of species richness of mangrove potamidids has been published. After compiling species distributions for both groups from taxonomic revisions and recent publications to compare patterns of species richness in mangrove potamidids and littorinids, it is clear that the highest diversity in both of these groups is in the Strait of Malacca and South China Sea, outside the boundaries of the Coral Triangle (Fig. 4B,C).

## Discussion

The highest diversity of mangrove plants is in the Indo-West Pacific, but the reported peak of this diversity differs between studies. Central and eastern Indonesia and Papua New Guinea are a highly diverse region, but it has also been reported that plants strictly restricted to mangrove forests and characterized by unique morphological and physiological adaptations may reach their highest diversity in the Strait of Malacca, at the western end of the Indo-Malay Archipelago^78^, or more broadly in Malaysia and Indonesia^79^. The existence of a peak of diversity for both the mangrove flora and mangrove gastropods in the riverine mangroves of the Strait of Malacca can be explained by the geological history of sea level changes in the Indo-West Pacific, which have played a large role in shaping the biogeography of the region, with the Sunda Shelf (i.e., Sumatra, Borneo, part of Java, and the area in between these islands) being mostly above sea level from the late Cretaceous to the early Miocene^80^. Onchidiid genera now living in mangrove forests are estimated to have emerged during this period, beginning approximately 46 million years ago during the Eocene^46^. In the early Miocene (ca. 20 Mya), marine waters began to penetrate parts of the eastern Sunda Shelf in what is now the Java Sea, as well as from the west over most of Sumatra^80^. However, the Sunda Shelf continued to serve as a barrier between the Indian Ocean, the South China Sea, and the Java Sea, and their respective marine fauna, and likely contributed to reduced dispersal of larvae between these regions, particularly during the Plio-Pleistocene glaciations when sea levels were between 60 and 120 m lower than present^81,82^.

Changing sea levels over thousands to millions of years led to dynamic changes in mangrove distributions during glacial cycles, with mangrove forests moving up and down the shore tracking sea levels^83,84^, but also facing local eradication on some oceanic islands during rapid sea-level rise^66,85^. The Strait of Malacca would only have connected the Indian Ocean and the Pacific Ocean during periods of the Plio-Pleistocene with the highest sea levels (i.e., with sea levels not lower than approximately 20 m below present), with mangroves shifting out of the strait to the edges of the shelf during glacial periods with lower sea levels^81^. Slugs and other animals were then isolated in mangroves on either side of this shelf for thousands of years. Given the short generation time of slugs and snails, with temperate *Onchidella* species laying egg capsules once or twice per year and quickly reaching adulthood, this is equivalent to thousands of generations of separation^45,49^. As sea levels rose, mangroves colonized the Strait of Malacca as it formed, bringing species from the Bay of Bengal and the South China Sea into contact.

The high species richness in riverine mangrove onchidiids in the Strait of Malacca, including Singapore at its eastern end, is partially due to the overlap in faunas from these two regions, similar to the Center of Overlap Hypothesis proposed to explain high levels of diversity in the Indo-West Pacific more broadly^86,87^. In fact, some onchidiid species living in riverine mangroves are distributed in the Strait of Malacca and the South China Sea (e.g., *Melayonchis annae*), others in the Strait of Malacca and the Bay of Bengal (e.g., *Onchidium melakense*, *Peronina tenera*, *Platevindex aptei*), while others are found from the Bay of Bengal through the Strait of Malacca to the South China Sea (e.g., *M. eloisae*, *O. stuxbergi*)^51,54,55,57^. However, the high species richness of onchidiids in the riverine mangroves of the Strait of Malacca is also due to the presence of species that are endemic to the strait, all of which were discovered during the recent systematic revision of the Onchidiidae, such as *Peronina zulfigari* and *Melayonchis tillieri*^52,57^.

Mangrove forests in the Indo-West Pacific are complex ecosystems with relatively low diversity compared to coral reefs in the region, but they are characterized by unique taxa specialized to survive harsh environmental conditions. Onchidiid slugs have become adapted to a variety of microhabitats inside riverine mangroves and are among the most diverse families of marine invertebrates in mangrove forests^46^. Species richness in onchidiids and two other groups of gastropods that have diversified in mangroves show that the highest diversity is outside the Coral Triangle, i.e., in the Strait of Malacca and South China Sea, indicating that the peak of species diversity of mangrove slugs in the Strait of Malacca and the South China Sea actually characterizes several unrelated mangrove gastropod taxa (Fig. 4).

The most diverse family of gastropods in mangrove forests are the ellobiid snails^88,89^, another group of pulmonates closely related to onchidiids, but unfortunately their systematics is completely outdated and unreliable and the diversity of this group has not been adequately surveyed in Southeast Asia^90^. Biogeographic analyses ought to be based on sound systematics as well as broad geographic sampling, and the ellobiid species names in the literature should not be trusted to test whether a similar peak of ellobiid diversity exists in the Strait of Malacca. As a result, the number of taxa that can be used for mangrove biogeography based on the data currently available is still quite limited. The data available for onchidiids represent one of the most diverse clades, and are of unprecedented quality in terms of both taxonomy and geographic coverage. These data indicate that the highest biodiversity of mangrove slugs occurs in the Strait of Malacca and South China Sea for, and that this pattern is shared by other groups of mangrove gastropods.

Further efforts to study the biodiversity of mangrove taxa are needed to enhance our understanding of biogeographic patterns in the Indo-West Pacific, but also most urgently because this fauna is threatened by the continued degradation and loss of mangrove forests^15,91–93^. Mangrove forests have declined due to extensive deforestation, but the mangrove plants themselves are also threatened, with 16% of true mangrove plant species (restricted to tropical intertidal habitats) considered threatened by the IUCN. This includes species in the Indo-West Pacific, such as *Sonneratia griffithii,* a species isolated to parts of India and Southeast Asia where 80% of mangrove area has been lost, which is listed as Critically Endangered^94^. Upstream, riverine and high intertidal mangroves tend to be more threatened by habitat loss than fringe mangroves, as they are often in close proximity to villages and the first to be cleared for timber and palm oil plantations, as well as the construction of aquaculture ponds^94,95^. Although there have been increasing efforts to restore mangrove forests over the last few decades, and reports of large restoration efforts, studies show that restored mangroves have lower biodiversity, stability, and ecosystem function compared to natural mangrove stands and that the ecosystem function of these restored mangroves is comparable to degraded mangroves for a long period of time^93,96^. Some of this depends on the restoration method used and the time since restoration, with a common method of replanting large stands of mangrove from a single species or even from a fast-growing species not native to the region, being a short-sighted approach to increase mangrove area. This is problematic especially when restoration is being done while destruction of natural mangroves occurs in other areas^97^. In our surveys of young, restored mangroves, onchidiids were either completely absent or occasionally present, but with never more than a single species. These are very low levels of diversity comparable to what we observed in mangroves heavily impacted by marine plastic pollution, where plastic debris brought in from rivers or the tides settles around the roots of mangrove trees, covering much of the sediment where gastropods feed (Goulding & Dayrat, unpublished). Successful methods have been developed to restore mangrove forests^97–100^, but successful restoration efforts require careful planning and are not an alternative to conserving natural mangrove stands and the unique diversity within them.

## Methods

### Taxon sampling and molecular markers

Geographic distributions of 64 onchidiid species sampled at 322 localities are used to estimate species richness globally, of which the distributions of 61 species are based on DNA sequences published in a series of taxonomic monographs by the authors revising the systematics of each genus^46,50–62^. DNA sequence data were generated from a large number of specimens in these integrative taxonomic studies because most onchidiid species cannot be distinguished from closely related species externally. Following the clustering of specimens based on analyses of mitochondrial and nuclear DNA sequences, anatomical examinations of each species were done, and additional museum specimens could be identified. Geographic records from China and Japan are included in the geographic distributions of species based on GenBank sequences that cluster with the taxa identified in our taxonomic studies^101–104^. Geographic distributions of the onchidiids outside the Indo-West Pacific are detailed in Goulding et al.^46^. The distribution of three species with no DNA sequence data are based on examination of museum specimens (*Platevindex latus* from New Ireland, Papua New Guinea, and *Onchidella lesliei* and *Onchidella steindachneri* from the Galapagos Islands)^54,105^. Species richness in Western Australia is estimated based on examination of museum specimens from the Australian Museum and the Western Australia Museum. References for the geographic distribution of each species are provided in Supplementary Table 1.

The geographic distribution of each species was created as a polygon in Google Earth Pro (https://www.google.com/earth/versions/#earth-pro) and exported as a KML file. One KML file included all marine onchidiid species (i.e., it did not include the terrestrial onchidiid *Semperoncis montana*). This KML file included 2 subdirectories based on primary habitat: one folder included 30 riverine mangrove species (all species in the genera *Alionchis*, *Laspionchis*, *Melayonchis*, *Onchidina*, *Onchidium*, *Paromoionchis*, *Peronina*, and *Platevindex*) and a second folder included 34 species found in the rocky intertidal and on coral rubble, and species found in fringe, coastal mangroves (all species in the genera *Marmaronchis*, *Onchidella*, *Peronia*, and *Wallaconchis*). A heat map was produced from these species distributions following the methods of Honeycut, 2012^106^. Species distribution polygons from each folder were imported into ArcMap v. 10.8.1 using the system conversion tool KML to Layer, and the polygon data was exported to a shape file. The Geoprocessing tool Union was used to calculate the number of overlapping distributions in polygons and generate a new feature class. The tool Multipart to Singlepart was used to create a new feature layer used as input for the tool Spatial Join with the match option “are identical to”. The overlap of species distributions in this layer was visualized by changing the layer properties. Under layer symbology, the overlap in each layer was evaluated with “Join Count” and graduated colors. In order to accommodate the large number of polygons, in the tab to edit manual classification of classes the maximum sample size was increased from 10,000 to 100,000. The transparency of the polygons was edited under display properties so that the spatial relationship of the polygon layers to the islands on the basemap underneath them could be visualized.

### Maps of other mangrove gastropods

Geographic distributions of potamidid species associated with mangroves were produced by reviewing geographic data published in taxonomic revisions. The geographic distributions of *Telescopium* and *Terebralia* are based on records published in a taxonomic study by Houbrick^107^, and the distributions of three other genera associated with mangroves (*Cerithidea, Cerithideopsis, Pirenella*) are from a series of taxonomic revisions and molecular phylogenetic studies published over the last 10 years^88,108–111^. We mapped the geographic data for each species of mangrove *Littoraria* based on the geographic records in Reid, 1986^77^ and updated these based on new geographic records he published and new data from recently described species^112–114^. Polygons representing the geographic distribution of each potamidid and littorinid were created in Google Earth and exported as a KML file.

The KML file for each gastropod family was imported into ArcGIS Pro 3.1.0. An analysis of overlap between species distributions was conducted in ArcGIS Pro, the same as conducted in ArcMap for the onchidiid distributions. In ArcGIS Pro, this analysis is straightforward and entailed using only the Geoprocessing tool “Count Overlapping Features” with the polygon data from the KML file as input.

### Figures

The blank maps used to create each figure were created using the program QGIS Desktop v.3.14.0 with the Natural Earth packages (https://www.naturalearthdata.com/). The maps illustrating the area of interest (either global or Indo-West Pacific) were exported, and then opened in Affinity Designer v. 2.1.1 to add text or illustrate patterns of species richness shown in the GIS analyses (https://affinity.serif.com/en-us/designer/).

### Supplementary Information

Supplementary Information.

## Data Availability

The KML file with all species distributions is available from https://doi.org/10.5061/dryad.fn2z34tzm.
