# Supplementary material for: The Coral Triangle and Strait of Malacca are two distinct hotspots of mangrove biodiversity
Source: Sci Rep. 2023 Sep 22;13:15793. doi: 10.1038/s41598-023-42057-6 (PMC10516942; doi:10.1038/s41598-023-42057-6)
Supplement: Supplementary file 1 — Supplementary Information. [file 41598_2023_42057_MOESM1_ESM.docx]

**Supplementary Table 1.** Onchidiid species categorized by habitat with references to data used to determine the geographic range of each species. Note: Identification of DNA sequences to species follows the taxonomic revisions of each genus published by the authors.

| **Species in riverine mangrove forests** | **Species in fringing mangroves and rocky intertidal** |
| --- | --- |
| *Alionchis jailoloensis* Goulding & Dayrat, 2018 ^1^ | *Marmaronchis marmoratus* (Lesson, 1831) ^2^ |
| *Laspionchis boucheti* Dayrat & Goulding, 2019 ^3,4^ | *Marmaronchis vaigiensis* (Quoy & Gaimard, 1825) ^2^ |
| *Laspionchis bourkei* Dayrat & Goulding, 2019 ^3^ | *Onchidella binneyi* Stearns, 1894 ^5,6^ |
| *Melayonchis aileenae* Dayrat & Goulding, 2017 ^7,8^ | *Onchidella celtica* (Cuvier *in* Audouin and Milne-Edwards, 1832) ^6^ |
| *Melayonchis annae* Dayrat, 2017 ^7,8^ | *Onchidella carpenteri* (Binney, 1861) ^6^ |
| *Melayonchis eberlyii* Dayrat & Goulding, 2019 ^8^ | *Onchidella floridana* (Dall, 1885) ^6^ |
| *Melayonchis eloisae* Dayrat, 2017 ^7,8^ | *Onchidella hansi* (Ev. Marcus and Er. Marcus, 1967) ^5,6^ |
| *Melayonchis siongkiati* Dayrat & Goulding, 2017 ^7,8^ | *Onchidella hildae* Hoffmann, 1928 ^6^ |
| *Melayonchis tillieri* Dayrat & Goulding, 2019 ^8^ | *Onchidella incisa* (Quoy and Gaimard, 1832) ^6^ |
| *Onchidina australis* (Semper, 1880) ^9^ | *Onchidella kurodai* Taki, 1935 ^6^ |
| *Onchidium melakense* Dayrat & Goulding, 2019 ^10^ | *Onchidella lesliei* (Stearns, 1892) ^5^ |
| *Onchidium reevesii* (J.E. Gray, 1850) ^10–12^ | *Onchidella maculata* Plate, 1893 ^6^ |
| *Onchidium stuxbergi* (Westerlund, 1883) ^10–12^ | *Onchidella marginata* (Couthouy *in* Gould, 1852) ^6,13^ |
| *Onchidium typhae* Buchannan, 1800 ^10,11^ | *Onchidella nigricans* (Quoy and Gaimard, 1832) ^6,14^ |
| *Paromoionchis boholensis* Dayrat & Goulding, 2019 ^15^ | *Onchidella steindachneri* (Semper, 1885) ^5^ |
| *Paromoionchis daemeli* (Semper, 1880) ^15^ | *Peronia griffithsi* Dayrat & Goulding, 2020 ^16^ |
| *Paromoionchis goslineri* Dayrat & Goulding, 2019 ^15^ | *Peronia madagascariensis* (Labbé, 1934) ^16^ |
| *Paromoionchis penangensis* Dayrat & Goulding, 2019 ^15^ | *Peronia okinawensis* Dayrat & Goulding, 2020 ^16^ |
| *Paromoionchis tumidus* (Semper, 1880) ^15^ | *Peronia peronii* (Cuvier, 1804) ^16^ |
| *Peronina tenera* (Stoliczka, 1869) ^17^ | *Peronia platei* (Hoffmann, 1928) ^16^ |
| *Peronina zulfigari* Goulding & Dayrat, 2018 ^17^ | *Peronia setoensis* Dayrat & Goulding, 2020 ^16^ |
| *Platevindex amboinae* (Plate, 1893) ^18^ | *Peronia sydneyensis* Dayrat & Goulding, 2020 ^16^ |
| *Platevindex applanatus* (Simroth, 1920) ^18^ | *Peronia verruculata* (Cuvier, 1830) ^12,16^ |
| *Platevindex aptei* Goulding & Dayrat, 2021 ^18^ | *Peronia willani* Dayrat & Goulding, 2020 ^16^ |
| *Platevindex burnupi* (Collinge, 1902) ^18^ | *Wallaconchis achleitneri* Goulding, 2018 ^19^ |
| *Platevindex coriaceus* (Semper, 1880) ^12,18,20^ | *Wallaconchis ater* (Lesson, 1830) ^19,20^ |
| *Platevindex latus* (Plate, 1893) ^18^ | *Wallaconchis buetschlii* (Stantschinsky,  1907) ^19^ |
| *Platevindex luteus* (Semper, 1880) ^18^ | *Wallaconchis comendadori* Goulding & Dayrat, 2018 ^19^ |
| *Platevindex martensi* (Plate, 1893) ^18,21^ | *Wallaconchis gracilis* (Stantschinsky,  1907) ^19^ |
| *Platevindex tigrinus* (Stoliczka, 1869) ^12,18^ | *Wallaconchis graniferus* (Semper, 1880) ^4,12,19^ |
|  | *Wallaconchis melanesiensis* Goulding & Dayrat, 2018 ^19,20^ |
|  | *Wallaconchis nangkauriensis* (Plate, 1893) ^19^ |
|  | *Wallaconchis sinanui* Goulding & Dayrat, 2018 ^19^ |
|  | *Wallaconchis uncinus* Goulding & Dayrat, 2018 ^19^ |

**References**

1. Goulding, T. C., Khalil, M., Tan, S. H. & Dayrat, B. A new genus and a new species of onchidiid slugs from eastern Indonesia (Gastropoda: Euthyneura: Onchidiidae). *Raffles Bull. Zool.* **66**, 337–349 (2018).

2. Dayrat, B., Goulding, T. C., Khalil, M., Lozouet, P. & Tan, S. H. Systematic revision one clade at a time: A new genus of onchidiid slugs from the Indo-West Pacific (Gastropoda: Euthyneura: Pulmonata). *Raffles Bull. Zool.* **66**, 814–837 (2018).

3. Dayrat, B. *et al.* A new genus of air-breathing marine slugs from South-East Asia (Gastropoda, Pulmonata, Onchidiidae). *Zookeys* **877**, 31–80 (2019).

4. Bravo, H. *et al.* A DNA barcode library for mangrove gastropods and crabs of Hong Kong and the Greater Bay Area reveals an unexpected faunal diversity associated with the intertidal forests of Southern China. *BMC Ecol. Evol.* **21**, 1–15 (2021).

5. Dayrat, B., Zimmermann, S. & Raposa, M. Taxonomic revision of the Onchidiidae (Mollusca: Gastropoda: Pulmonata) from the Tropical Eastern Pacific. *J. Nat. Hist.* **45**, 939–1003 (2011).

6. Goulding, T. C., Khalil, M., Tan, S. H. & Dayrat, B. Global diversification and evolutionary history of onchidiid slugs (Gastropoda, Pulmonata). *Mol. Phylogenet. Evol.* **168**, 1–22 (2022).

7. Dayrat, B., Goulding, T. C., Apte, D., Bhave, V. & Quảng, N. X. A new genus and four new species of onchidiid slugs from South-East Asia (Mollusca: Gastropoda: Pulmonata: Onchidiidae). *J. Nat. Hist.* **51**, 1851–1897 (2017).

8. Dayrat, B., Goulding, T. C., Bourke, A. J., Khalil, M. & Tan, S. H. New species and new records of *Melayonchis* slugs (Gastropoda : Euthyneura : Pulmonata : Onchidiidae). *Raffles Bull. Zool.* **67**, 557–585 (2019).

9. Dayrat, B. & Goulding, T. C. Systematics of the onchidiid slug *Onchidina australis* (Mollusca: Gastropoda: Pulmonata). *Arch. für Molluskenkd.* **146**, 121–133 (2017).

10. Dayrat, B., Goulding, T. C., Khalil, M., Apte, D. & Tan, S. H. A new species and new records of *Onchidium* slugs (Gastropoda, Euthyneura, Pulmonata, Onchidiidae) in South-East Asia. *Zookeys* **2019**, 27–57 (2019).

11. Dayrat, B. *et al.* Integrative taxonomy of the genus *Onchidium* Buchannan, 1800 (Mollusca: Gastropoda: Pulmonata: Onchidiidae). *Zookeys* 1–40 (2016).

12. Sun, B. *et al.* Species diversity of Onchidiidae (Eupulmonata: Heterobranchia) on the mainland of China based on molecular data. *Molluscan Res.* **34**, 62–70 (2014).

13. Cumming, R. A., Nikula, R., Spencer, H. G. & Waters, J. M. Transoceanic genetic similarities of kelp-associated sea slug populations: Long-distance dispersal via rafting? *J. Biogeogr.* **41**, 2357–2370 (2014).

14. Cumming, R. A., Nikula, R., Spencer, H. G. & Waters, J. M. Trans-Tasman genetic connectivity in the intertidal air-breathing slug Onchidella nigricans. *Mar. Ecol. Prog. Ser.* **562**, 93–100 (2016).

15. Dayrat, B. *et al.* A new genus and three new species of mangrove slugs from the Indo- West Pacific (Mollusca: Gastropoda: Euthyneura: Onchidiidae). *Eur. J. Taxon.* **500**, 1–77 (2019).

16. Dayrat, B. *et al.* Systematic revision of *Peronia*, Fleming 1822 (Mollusca: Gastropoda: Pulmonata: Onchidiidae). *Zookeys* **972**, 1–224 (2020).

17. Goulding, T. C. *et al.* A revision of *Peronina* Plate, 1893 (Gastropoda: Euthyneura: Onchidiidae) based on mitochondrial and nuclear DNA sequences, morphology, and natural history. *Invertebr. Syst.* **32**, 803–826 (2018).

18. Goulding, T. C. *et al.* Systematic revision of *Platevindex* Baker, 1938 (Gastropoda: Euthyneura: Onchidiidae). *Eur. J. Taxon.* **737**, 1–133. (2021).

19. Goulding, T. C., Khalil, M., Tan, S. H. & Dayrat, B. Integrative taxonomy of a new and highly-diverse genus of onchidiid slugs from the Coral Triangle (Gastropoda: Pulmonata: Onchidiidae). *Zookeys* **763**, 1–111 (2018).

20. Takagi, M., Takao, Y., Mizuno, K. & Ieyama, H. Genetic diversity of Onchidiidae in Japan. *Fauna Ryukyuana* **49**, 23–37 (2019).

21. Chen, C., Shen, H. D. & Wang, L. Systematic Classification of Onchidiidae (Mollusca: Gastropoda: Pulmonata) in Mainland China Based on Three Genes. *Unpublished*.
